# Optimizing nanomaterial dosages in concrete for structural applications using experimental design techniques

**DOI:** 10.1038/s41598-025-05265-w

**Published:** 2025-07-01

**Authors:** Ratchagaraja Dhairiyasamy, Deepika Gabiriel, Deekshant Varshney, Subhav Singh

**Affiliations:** 1 Saveetha School of Engineering, Department of Electronics and Communication Engineering, Saveetha Institute of Medical and Technical Sciences, Saveetha University, Chennai, Tamil Nadu India; 2 College of Engineering and Technology, Aksum University, Aksum, Ethiopia; 3 Centre of Research Impact and Outcome, Chitkara University Institute of Engineering and Technology, Chitkara University, Rajpura, 140417 Punjab India; 4 Chitkara Centre for Research and Development, Chitkara University, Shimla, 174103 Himachal Pradesh India; 5 Division of Research and Development, Lovely Professional University, Phagwara, Punjab India; 6 Centre for Promotion of Research, Graphic Era (Deemed to be University), Dehradun, 248002 Uttarakhand India; 7 Division of Research & Innovation, Uttaranchal University, Dehradun, India

**Keywords:** Nano-silica, Nano-alumina, Graphene oxide, Concrete, Mechanical properties, Durability, Microstructure, Engineering, Materials science

## Abstract

Nanomaterial-enhanced concrete offers a transformative route to improve mechanical and durability performance for structural applications. Despite the promise of nano-silica (NS), nano-alumina (NA), and graphene oxide (GO), a comparative evaluation under unified conditions remains limited. This study addresses that gap by experimentally investigating the effects of NS (1–3%), NA (1–3%), and GO (0.05–0.15%) on workability, strength, and durability properties of concrete. A two-factor statistical optimization using Response Surface Methodology (RSM) was employed to model and predict compressive, tensile, and flexural strengths as functions of nanomaterial and superplasticizer dosages. Experimental results showed that NS and GO achieved a ~ 25% increase in compressive strength, while GO yielded the highest flexural improvement (~ 40%) at only 0.10% dosage. Durability metrics such as RCPT charge passed, water absorption, and sulfate resistance were significantly enhanced across all nano-modified mixes, with NS and GO outperforming NA. RSM confirmed nanomaterial dosage as the dominant factor influencing strength, while superplasticizer had no statistically significant effect. Optimal dosages were identified for each nanomaterial to maximize performance while avoiding overdosing effects. This study provides a comprehensive, optimization-driven comparison of NS, NA, and GO in concrete, offering valuable insights for designing durable, high-performance cementitious systems using tailored nanomodification strategies.

## Introduction

Concrete is the most widely used construction material, but its performance is limited by inherent brittleness, porosity, and vulnerability to durability challenges. Recent advancements in nanotechnology have enabled the use of nanomaterials to tailor the microstructure of concrete and significantly enhance its mechanical and durability properties. Nano-sized mineral admixtures such as nano-silica (NS) and nano-alumina (NA), as well as carbon-based nanomaterials like graphene oxide (GO), have shown promise in improving compressive Strength, tensile/flexural behavior, and long-term durability of cementitious composites^1^. NS (SiO_2_ nanoparticles) is a highly reactive pozzolanic material that can consume calcium hydroxide and form additional C–S–H gel, refining the pore structure and strengthening the cement paste. NA (Al_2_O_3_ nanoparticles) primarily acts as a micro-filler and nucleation site, accelerating hydration and densifying the matrix, which can particularly improve early-age strength and reduce porosity. GO, an oxidized form of graphene with atomic thickness and high aspect ratio, introduces a two-dimensional nanostructure that can bridge micro-cracks and provide nano-reinforcement, as well as serve as nucleation sites for hydration products, leading to a more crack-resistant and impermeable concrete^2^.

Several studies have independently reported the benefits of these nanomaterials. For example, adding a small percentage of NS has been shown to markedly increase compressive strength and reduce chloride permeability due to its pore-filling and secondary hydration effects^3^. NA has been found effective in increasing compressive strength at optimal dosages (around 1%), although excessive NA can lead to agglomeration and diminish workability, limiting further gains. GO has attracted attention for its ability to improve flexural and tensile strength disproportionately to its dosage, owing to its ability to arrest crack propagation and enhance the toughness of the cement matrix. Despite these advancements, direct comparisons of NS, NA, and GO under similar mix design conditions are limited^4^. Each nanomaterial has a distinct mechanism of action and optimal dosage range, and an understanding of their relative efficacy is important for selecting the appropriate additive for a given structural performance goal.

**Table 1:** Nanomaterial-modified concrete and their relevance to the present study.

Materials studied	Concrete type	Focus area	Methodology	Relevance to current study
Micro-silica, Nano- silica, Fly ash	Modified OPC concrete	Strength & Microstructure	Mechanical tests, SEM	Compared NS + micro fillers; no RSM or GO^5^
Nano-silica	High-temp resistant concrete	High temp resistance & microcracks	TGA, SEM, porosimetry	Focus on heat resistance only; single nano ^6^
Stabilized nano-silica, Silica fume	High performance concrete	Dispersion and strength gain	SEM, modulus test	Improved Strength with NS; no GO or NA^7^
Interface zone (SCI)	Reinforced concrete	SCI prep & nano- mechanics	SEM, Nano- indentation	SCI analysis only; no material comparison^8^
Nano-SiO_2_, Nano-Al_2_O_3_	Geopolymer concrete	Kinetics & microstructure in geopolymer	XRD, SEM, JMAK model	In geopolymer, not OPC; limited comparability^9^
Nano-TiO_2_	Reactive powder concrete (RPC)	Strength & conductivity	XRD, SEM, electrical tests	Only NT studied; no NS/NA/GO contrast^10^
Nano-graphene	Autoclaved aerated concrete	Strength, impact, and absorption	SEM, XRD, mechanical	Specialty AAC product; not conventional concrete^11^
UHPC, Heat curing	Ultra-high-performance concrete	Curing effects on microstructure	Nano-indentation, XRD, SEM	Curing focus in UHPC, no dosage optimization^12^
Nano-CaCO_3_	UHPC with steel fibers	Fiber bond & ITZ	BSEM, MIP, fiber pull-out	CaCO_3_ in UHPC, focus on fiber bond^13^
Nano-SiO_2_, Steel fiber	Steel fiber reinforced concrete	High-temp mechanicals, ITZ	XRD, SEM, HT testing	NS in SFRC; thermal focus, not multi- nano^14^
Nano-silica, Basalt fiber	Recycled concrete	Elevated temp performance	XRD, SEM	Uses NS + fiber in recycled mix; not mechanical-durability balance^15^
Nano-silica on RAC	Recycled aggregate concrete	RAC enhancement with NS treatment	SEM, physical pre-treatment	RAC quality improved by NS; lacks NS vs. NA vs. GO study^16^

Although nanomaterials such as nano-silica (NS), nano-alumina (NA), and graphene oxide (GO) have been widely investigated in cementitious systems, previous studies have typically explored them in isolation, within non-standard matrices (e.g., geopolymers), or without fully examining their synergistic or comparative effects under identical experimental conditions. The literature (Table 1) often focuses on specific properties such as compressive strength or chloride resistance but lacks an integrated evaluation covering both mechanical and durability performance in a unified concrete matrix. Additionally, while optimization tools like (RSM) have been applied in select cases, very few studies incorporate RSM alongside physical experimentation to determine the optimal dosage of each nanomaterial for enhanced structural performance^17^.

Unlike the existing literature that tends to evaluate individual nanomaterials in isolation or under specialized conditions such as geopolymers, UHPC, or autoclaved systems, the present study offers a comprehensive, side-by-side comparison of nano-silica (NS), nano-alumina (NA), and graphene oxide (GO) within a conventional concrete matrix. By adopting a unified experimental framework with a consistent mix design, curing, and testing protocols, this research isolates the true performance characteristics of each nanomaterial^18^. Furthermore, the integration of RSM for dosage optimization across mechanical and durability parameters sets this work apart from previous studies that lacked such statistical refinement. This dual experimental–analytical approach provides actionable insights for the targeted selection and application of nanomaterials in structural concrete, establishing a new benchmark for comparative nanotechnology-driven concrete enhancement^19^.

This research addresses that gap by conducting a comprehensive, side-by-side experimental assessment of NS, NA, and GO in conventional concrete, using standardized proportions, curing protocols, and test procedures. Mechanical parameters, including compressive, splitting tensile, and flexural strengths were evaluated, alongside key durability metrics such as rapid chloride penetration (RCPT), water absorption, and sulfate resistance. The use of RSM further allowed for statistical optimization of dosage levels, highlighting non-linear relationships and identifying performance peaks for each nanomaterial. Microstructural analysis through SEM and TGA provided additional insight into the mechanisms governing observed improvements. The novelty of this work lies in its holistic, experimentally grounded comparison of three functionally distinct nanomaterials within a single framework—offering clear, data-driven guidance for high-performance, durable, and sustainable concrete design.

## Materials and methods

**Fig. 1:**
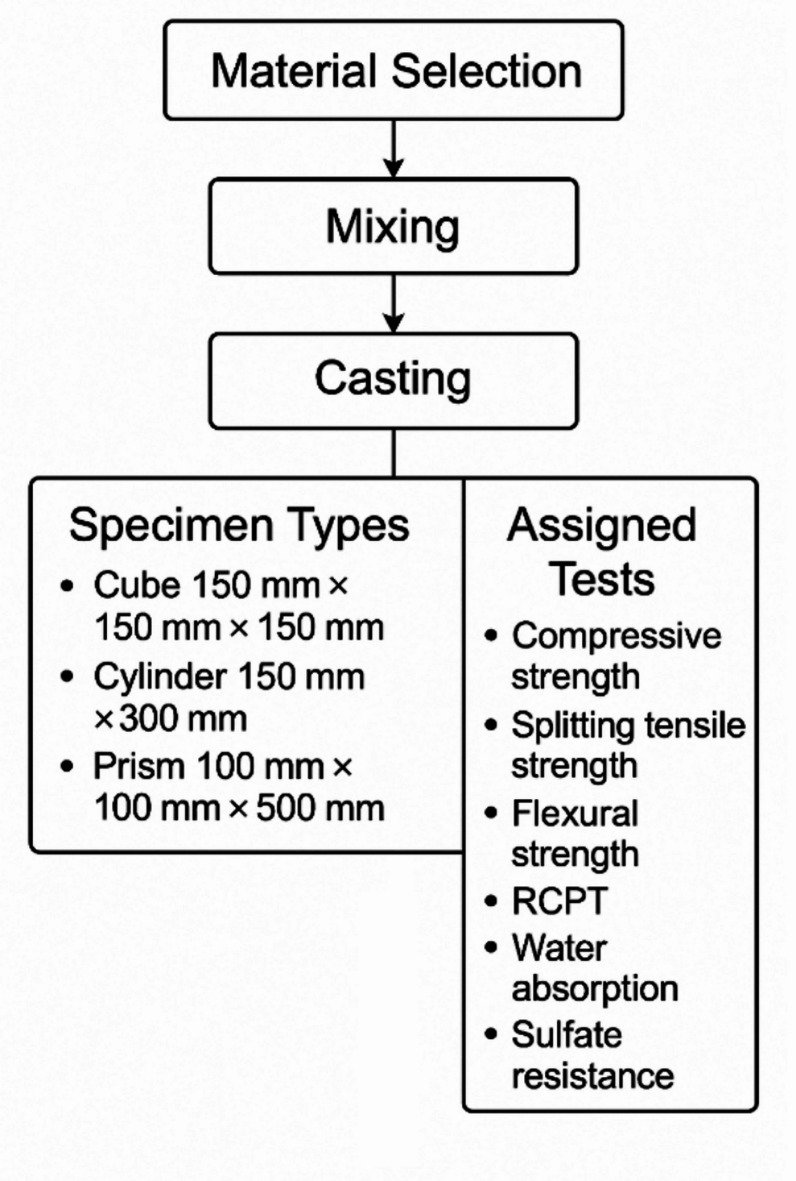
Flowchart of experiment.

A standard concrete mix design was adopted as the control baseline, and then modified with nano-silica, nano-alumina, or graphene oxide at various dosages. Ordinary Portland cement (OPC) (53 Grade, conforming to ASTM C150 Type I specifications) was used as the primary binder. Locally sourced river sand with a fineness modulus of about 2.7 was used as fine aggregate, and crushed granite with a maximum size of 20 mm served as coarse aggregate. The mix proportion for the control concrete (per 1 m^3^) was 1:1.75:3.0 (cement: sand: coarse aggregate by weight) with a water–cement ratio (w/c) of 0.40. This resulted in a cement content of approximately 400 kg/m^3^ and a water content of 160 kg/m^3^, yielding a target 28-day compressive strength of around 40 MPa for the control mix (Fig. 1).

Nano-silica (NS) in dry powder form (average particle size ~ 15 nm, > 99% SiO_2_ purity) was used as a partial replacement of cement by weight. Nano-alumina (NA) powder (particle size ~ 20–30 nm, > 99% Al_2_O_3_) was similarly used as a cement replacement. Graphene oxide (GO) was obtained as an aqueous suspension (with GO sheets on the order of a few micrometers lateral size and < 5 nm thickness); the GO solids content was incorporated as a percentage of cement mass. For each nanomaterial, three dosage levels were selected based on literature and preliminary tests: 1%, 2%, 3% by weight of cement for NS and NA, and 0.05%, 0.10%, 0.15% for GO (which correspond to approximately 1, 2, 3 kg of GO per cubic meter when expressed per cement weight%)^20^. In the NS and NA mixes, the nanoparticle mass replaced an equivalent mass of cement to keep the total binder content constant at 400 kg/m^3^. In GO mixes, due to the extremely small weight, the cement content was effectively unchanged (e.g. at 0.10% GO, 0.4 kg of cement would be replaced by GO per m^3^). A high range polycarboxylate superplasticizer (SP) was added at a dosage of 0.5% by cement weight to all mixes to improve workability; for GO mixes, a slightly higher SP dosage (up to ~ 1.0%) and vigorous mixing were used to ensure good dispersion of the GO.

**Fig. 2:**
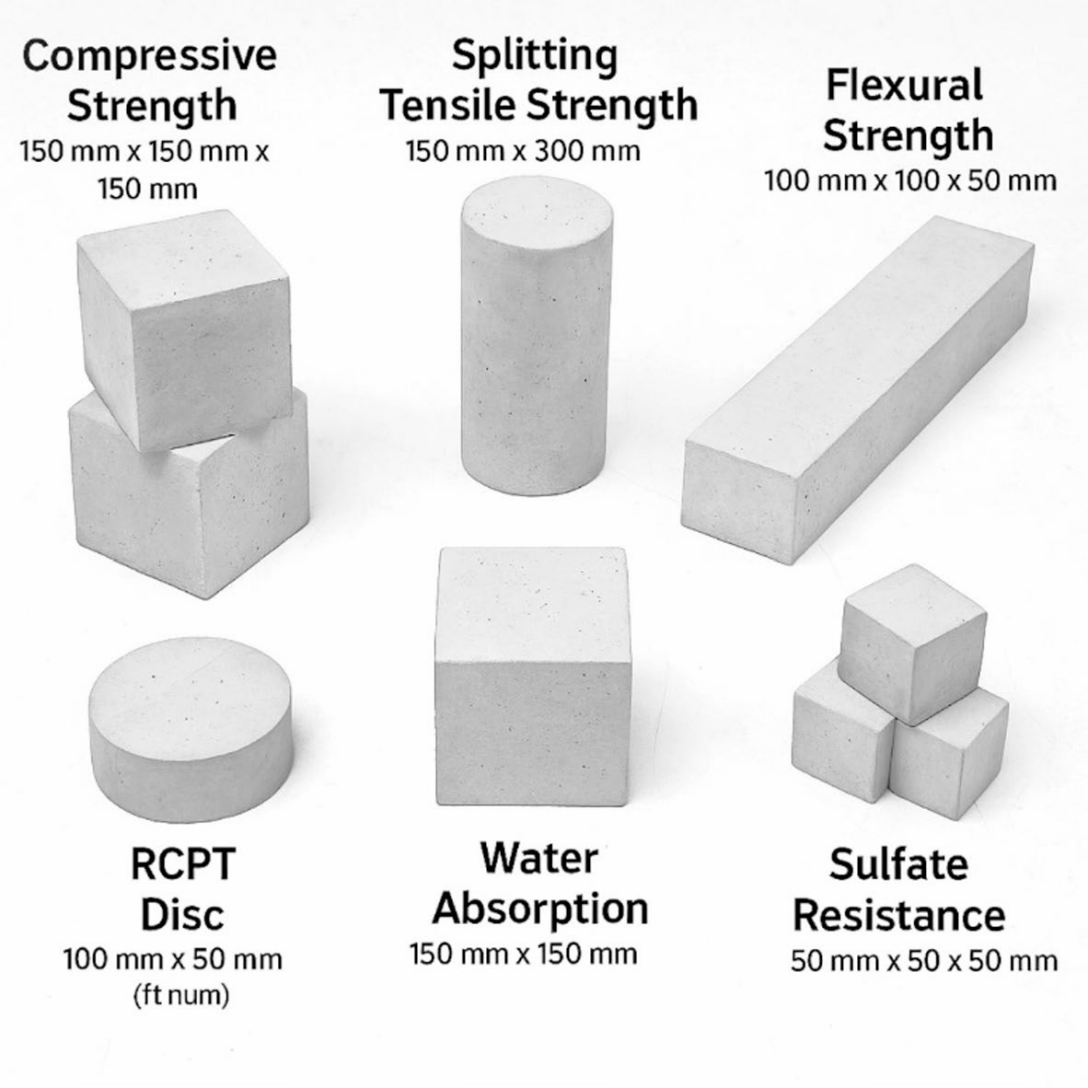
Prepared samples.

**Table 2:** Mix design proportions for 1 m^3^ of concrete (binder and aggregate quantities for control and nano-modified mixes).

Mix ID	Cement (kg)	NS (kg)	NA (kg)	GO (kg)	Water (kg)	w/c	Fine Agg (kg)	Coarse Agg (kg)
Control	400	0	0	0	160	0.40	700	1100
NS-1%	396	4.0	0	0	160	0.40	700	1100
NS-2%	392	8.0	0	0	160	0.40	700	1100
NS-3%	388	12.0	0	0	160	0.40	700	1100
NA-1%	396	0	4.0	0	160	0.40	700	1100
NA-2%	392	0	8.0	0	160	0.40	700	1100
NA-3%	388	0	12.0	0	160	0.40	700	1100
GO-0.05%	399.8	0	0	0.2	160	0.40	700	1100
GO-0.10%	399.6	0	0	0.4	160	0.40	700	1100
GO-0.15%	399.4	0	0	0.6	160	0.40	700	1100

Cement, NS, and NA contents are given in kg (per cubic meter) with nano-additive dosages expressed as % of cement. All mixes had water = 160 kg (w/c = 0.40), fine aggregate = 700 kg, coarse aggregate = 1100 kg, and superplasticizer = 0.5% of cement (Table 2). All dry materials (cement, sand, coarse aggregate, and NS/NA powders) were mixed in a pan mixer for 2 min to ensure uniform dispersion of the nanoparticles in the dry blend (Fig. 2). Table 3 includes all mechanical and durability tests performed in the study, such as compressive strength, splitting tensile Strength, flexural Strength, RCPT, water absorption, and sulfate resistance, along with their respective specimen sizes and shapes.

**Table 3:**
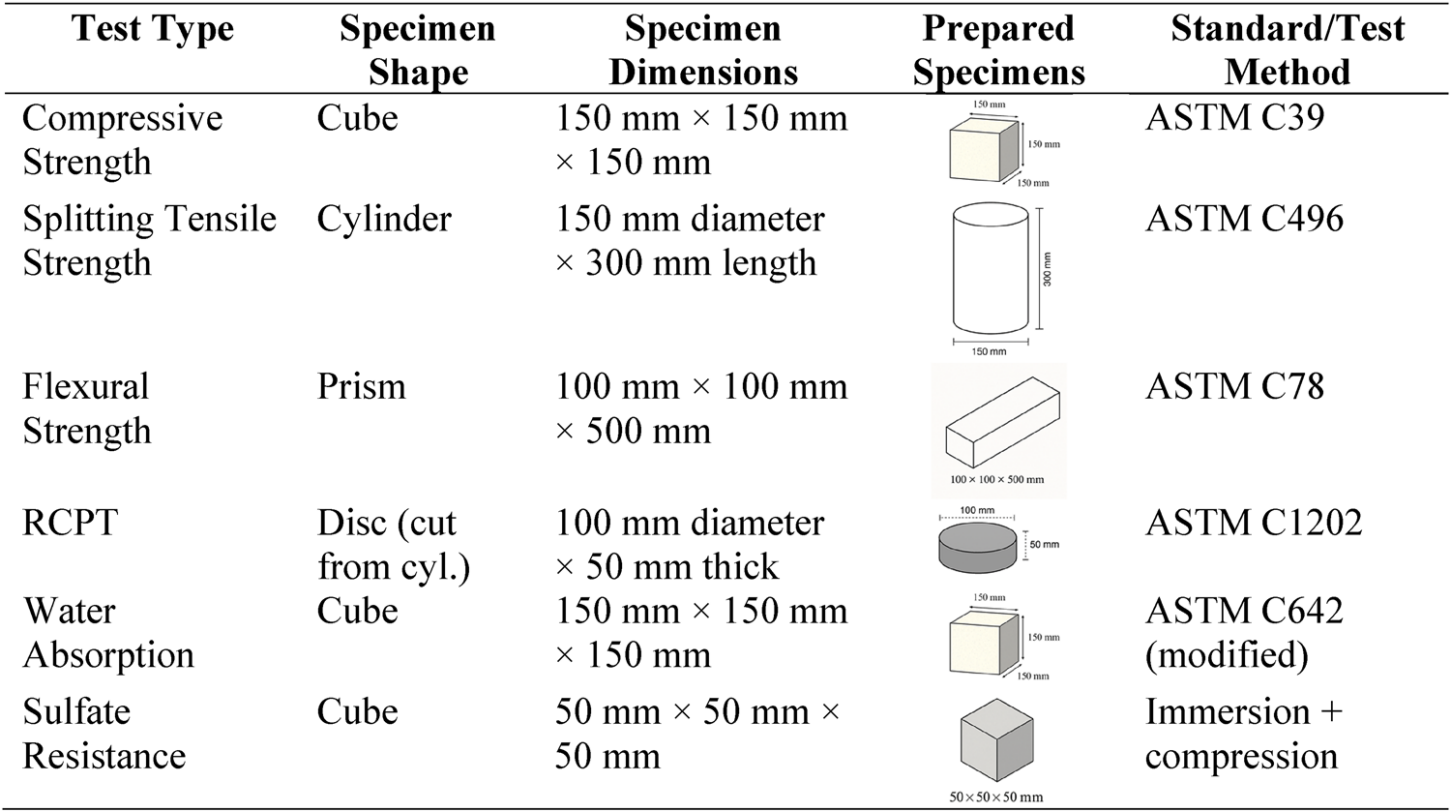
Tests and corresponding specimen types and sizes.

Water (with dissolved superplasticizer) was then added gradually and mixing continued for another 3–4 min. For GO mixes, the GO suspension was ultrasonicated for 30 min prior to mixing, and then it was added to the mixing water; an extended mixing time of ~ 5 min was used to promote even distribution of GO sheets throughout the paste^21^. The fresh concrete was checked for consistency and then cast into molds for testing. Standard 150 mm cubes were cast for compressive strength tests, 150 mm × 300 mm cylinders for splitting tensile tests, and 100 mm × 100 mm × 500 mm prisms for flexural strength tests. Specimens were compacted on a vibrating table to remove air voids and ensure good finish. After casting, all samples were covered with plastic sheets for 24 h and then demolded and cured in a water tank at 23 ± 2 C until the testing ages.

The slump cone test (ASTM C143) was conducted on the fresh concrete of each mix to assess workability. A standard Abrams cone was filled with concrete in three layers with rodding, and the subsidence (slump) of the cone upon lifting was measured in millimeters. Due to the inclusion of nanoparticles, which can significantly affect rheology, all mixes were tested for slump immediately after mixing. No additional water was added to adjust slump; only the fixed SP dosage was used, so the measured slump reflects the inherent workability of each formulation^22^.

Compressive strength was tested on 150 mm cube specimens at 7 and 28 days of curing (with the primary focus on 28-day results for comparison). The cubes were tested in a compression testing machine at a loading rate of 0.5 MPa/s until failure, and the maximum load was recorded to compute compressive strength. Splitting tensile strength was measured at 28 days on 150 × 300 mm cylindrical specimens following ASTM C496: each cylinder was loaded diametrically between plywood bearing strips at a rate producing failure in ~ 2 min, and the splitting tensile Strength was calculated from the peak load. Flexural Strength (modulus of rupture) was tested at 28 days using 100 × 100 × 500 mm prism specimens under four-point bending (per ASTM C78). The span length was 400 mm with loading pins placed at third points; load was applied at a rate of about 0.05 MPa/s tension stress in the bottom fiber. The maximum load at fracture was used to calculate the flexural strength. For each test and mix, at least three specimens were tested, and the average value was reported.

**Fig. 3:**
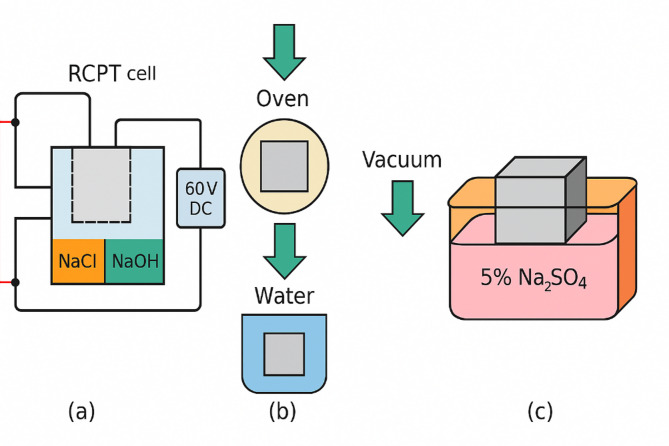
(a) RCPT, (b) Water Absorption, and (c) Sulfate Resistance Test Procedures.

Rapid chloride permeability tests (RCPT) were conducted in accordance with ASTM C1202 on 28-day water-cured specimens (Fig. 3). From each concrete, a 50 mm thick disk was cut from a 100 mm diameter cylindrical specimen. The sides of the disk were sealed, and the sample was placed in the RCPT cell with one side in contact with 3% NaCl solution and the other side with 0.3 N NaOH solution. A 60 V DC potential was applied across the cell for 6 h, and the total charge passed (in coulombs) was recorded, which indicates the concrete’s resistance to chloride ion penetration (lower charge = higher resistance). Water absorption was measured following a vacuum saturation method like ASTM C642. 28-day cured cubic specimens were oven-dried at 105 °C to constant mass, then immersed in water under vacuum for 24 h^23^. The percentage increase in mass (relative to dry mass) was recorded as the water absorption, reflecting the concrete’s porosity and water permeability. An immersion test evaluated sulfate resistance: 50 mm cube specimens (one from each mix) were immersed in a 5% sodium sulfate (Na_2_SO_4_) solution for 56 days after an initial 28-day curing period.

Another set of companion cubes was stored in water as control curing. After 56 days of sulfate exposure, the cubes were tested in compression. The strength retention percentage quantified the degree of sulfate attack, i.e. the compressive strength of sulfate-exposed specimens expressed as a percentage of the strength of the water-cured specimens. In addition, visual observation for cracking or surface deterioration and length change measurements were made to detect any expansion due to sulfate; however, no significant dimensional changes were noted over the 56 days given the moderate sulfate concentration and relatively short duration.

To investigate the internal structural modifications caused by the nanomaterials, samples of the hardened cement paste from the fracture surfaces of 28-day specimens were examined using (SEM). Small pieces (~ 10 mm fragments) were taken from the core of broken specimens (to avoid carbonation on outer surfaces), vacuum-dried, and gold-coated for SEM imaging. A JEOL high-resolution SEM was used to observe the morphology of hydration products, the distribution of nanoparticles, and the interfacial transition zones in each mix^24^. Energy dispersive X-ray spectroscopy (EDS) attached to the SEM was used qualitatively to verify the presence of silica or alumina enrichment in areas corresponding to NS or NA, and to detect any residues of GO (indirectly by carbon mapping, recognizing that GO may be largely consumed/embedded in hydrates). The focus was on comparing pore structure and crack patterns between the control and nano-modified concretes. Additionally, X-ray diffraction (XRD) analysis was conducted on powdered samples from each mix at 28 days to identify changes in crystalline phases (especially the quantity of portlandite (CH) peaks, which would be reduced in the presence of pozzolanic NS). Thermogravimetric analysis (TGA) was also performed on crushed paste samples to quantify the CH content by the weight loss between 400 and 500 °C; these results supported the extent of pozzolanic reaction in NS mixes compared to NA and GO mixes^25^.

## Results and discussion

**Fig. 4:**
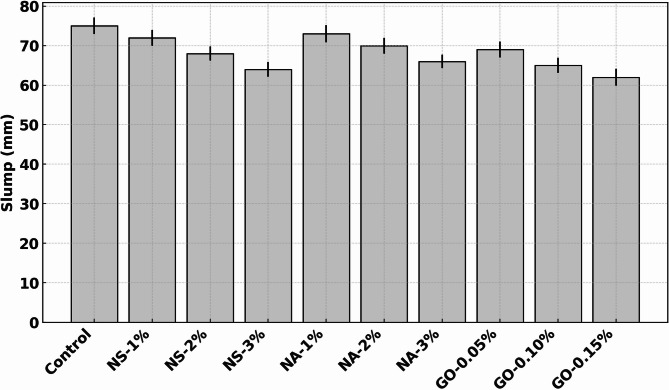
Effect of nanomaterial incorporation on concrete workability (slump).

Figure 4 shows the slump values of concrete mixes incorporating different dosages of nano-silica (NS), nano-alumina (NA), and graphene oxide (GO), highlighting the impact of nano-additives on workability. The control mix exhibited a slump of approximately 75 mm, indicating moderate flowability suitable for conventional placement methods. Incorporation of NS led to a progressive reduction in slump. At 1% NS, the slump reduced slightly to ~ 72 mm (~ 4% decrease), followed by more significant drops at 2% (~ 68 mm) and 3% (~ 63 mm), corresponding to ~ 9% and ~ 16% reductions, respectively^26^. This trend is attributed to the high surface area and strong water demand of NS particles, which absorb free water and increase inter-particle friction, thereby reducing mix fluidity. NA-modified concretes showed a similar but slightly less pronounced pattern^27^. NA-1% maintained good workability with a slump of ~ 73 mm (~ 3% reduction), but higher dosages at 2% and 3% resulted in ~ 70 mm and ~ 65 mm slumps, indicating ~ 7% and ~ 13% reductions from control. While NA has similar particle fineness, its dispersion behavior may be slightly more favourable at moderate dosages. GO-modified mixes also exhibited decreasing slump with increasing content. At 0.05% GO, the slump was ~ 69 mm (~ 8% decrease), reducing further to ~ 66 mm at 0.10% GO and ~ 62 mm at 0.15% GO (~ 17% reduction overall). GO’s flake-like morphology and high specific surface area increase the viscosity of the mix, leading to reduced flow. The addition of nanomaterials negatively affected workability, with higher dosages causing more pronounced reductions. Among them, NS and GO exhibited stronger water-demanding behavior, necessitating superplasticizer adjustments for practical applications. Proper dosage optimization is therefore crucial to balance workability and performance enhancements in nano-modified concrete.

### Workability

The fresh concrete remained cohesive without signs of segregation or excessive bleeding in all cases, which suggests that the nanoparticle additions did not adversely affect stability, only the consistency. The dosage of superplasticizer (SP) in each mix was determined through preliminary workability trials aiming to achieve cohesive mixes without segregation. For NS and NA mixes, a uniform SP dosage of 0.5% by cement weight was maintained to isolate the effect of nanoparticle incorporation on slump. However, GO mixes required a slightly increased SP dosage (0.8–1.0%) to counteract the elevated viscosity and particle agglomeration caused by GO’s high surface area and hydrophilic functional groups. Although a higher SP dosage could influence slump, it was necessary to ensure adequate dispersion of GO sheets and maintain casting feasibility^28^. The reported slump values therefore reflect both the rheological effect of the nanomaterials, and the admixture adjustment needed for homogeneity. As all SP dosages were kept within the recommended range and optimized individually, the variations in slump are considered representative of true material behavior, rather than being artifacts of inconsistent admixture dosing. The fresh property tests indicate a trade-off: the addition of NS, NA, or GO will require careful water/SP management to ensure sufficient workability for casting and compaction^29^. Compressive strengths are from cube tests and splitting tensile and flexural strengths are from cylinder and prism tests, respectively. Standard deviation for compressive strength was within ± 1.2 MPa (for modified mixes) and ± 0.8 MPa (control). All mixes showed improved strengths compared to control, with peak values at the indicated optimum dosages.

**Table 4:** Mechanical properties (mean values) for control and nano-modified concretes.

Mix ID	Compressive strength (MPa)	Splitting tensile (MPa)	Flexural strength (MPa)
Control	40.0	3.5	5.0
NS-1%	45.0	3.8	5.3
NS-2%	50.0	4.2	6.0
NS-3%	48.0	4.0	5.8
NA-1%	48.0	4.0	5.5
NA-2%	47.0	3.9	5.6
NA-3%	45.0	3.8	5.4
GO-0.05%	44.0	3.7	5.5
GO-0.10%	50.0	4.3	7.0
GO-0.15%	48.0	4.1	6.8

The 28-day mechanical test results for all mixes are summarized in Table 4. It is evident that the inclusion of NS, NA, or GO leads to an enhancement in all strength metrics relative to the control, although the extent of improvement depends on the type of nanomaterial and its dosage. Each additive exhibits an optimum dosage for maximizing strength, beyond which the benefits taper off or even slightly reverse due to possible negative effects like particle agglomeration or dilution of cement content.

### Compressive strength

**Fig. 5:**
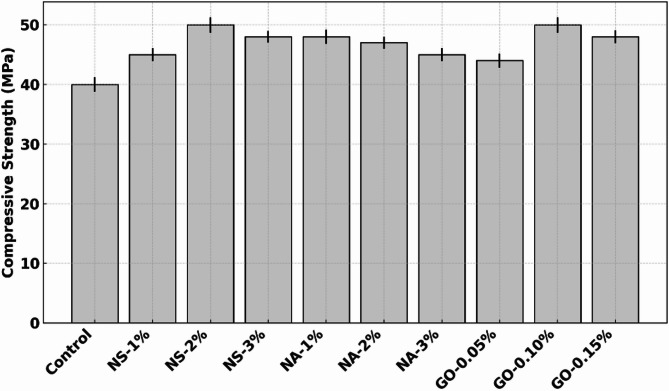
Compressive strength versus nanomaterial dosage.

Figure 5 shows the 28-day compressive strength of concrete with varying dosages of nano-silica (NS), nano-alumina (NA), and graphene oxide (GO) compared to a control mix. The control concrete achieved a compressive strength of approximately 40 MPa. Incorporation of NS significantly enhanced strength: at 1% NS, Strength increased to ~ 45 MPa (~ 12.5% rise) and peaked at 2% NS with ~ 50 MPa—a notable ~ 25% improvement. At 3% NS, Strength remained high at ~ 48 MPa, suggesting a slight reduction from the optimum, possibly due to particle agglomeration that impairs matrix uniformity. NA also showed strength enhancement. At 1% NA, compressive strength was ~ 48 MPa (~ 20% higher than control)^30^. However, with increasing dosages, performance declined slightly: ~47 MPa at 2% and ~ 45 MPa at 3%, indicating that while NA is effective, higher contents may disrupt the matrix due to poor dispersion or filler saturation, reducing hydration efficiency. GO showed a consistent upward trend. At 0.05%, compressive strength rose to ~ 44 MPa, increasing further to ~ 50 MPa at 0.10% GO—equal to NS-2%. At 0.15% GO, the strength was slightly lower (~ 48 MPa) but still markedly higher than the control^31^. The improvement in GO mixes is attributed to its ability to bridge microcracks, densify the microstructure, and act as nucleation sites for hydration products, enhancing overall strength. NS-2% and GO-0.10% demonstrated the highest strength gains (~ 25%), with NA-1% close behind. These results confirm that optimal nanomaterial dosages significantly improve compressive performance by refining pore structure, accelerating hydration, and enhancing matrix integrity. Overdosage, however, may reduce these benefits due to particle agglomeration or ineffective dispersion.

### Splitting tensile strength

**Fig. 6:**
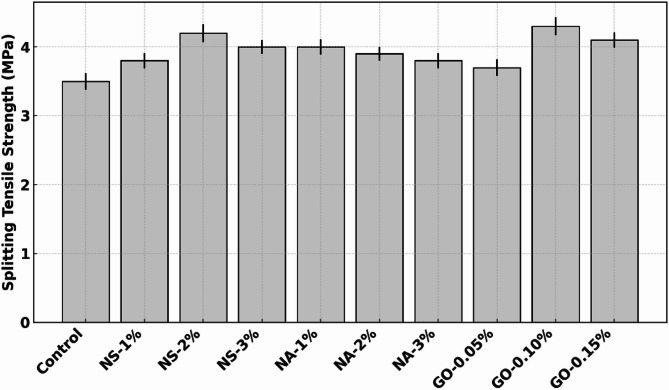
Splitting tensile strength as a function of nano-additive dosage.

Figure 6 shows the 28-day splitting tensile Strength of concrete modified with different dosages of nano-silica (NS), nano-alumina (NA), and graphene oxide (GO), in comparison to a control mix. The control exhibited a tensile strength of approximately 3.5 MPa. NS incorporation significantly enhanced performance: at 1% NS, tensile strength increased to ~ 3.8 MPa (~ 8.6% improvement) and further rise to ~ 4.2 MPa at 2% NS (~ 20% gain). At 3% NS, a slight drop to ~ 4.0 MPa was observed, indicating that while NS improves tensile capacity due to improved microstructure and bond strength, exceeding the optimal dosage may cause particle agglomeration and reduce efficiency. NA also contributed to tensile strength gains. NA-1% achieved ~ 4.0 MPa (~ 14.3% increase from control), but strength slightly declined at 2% (~ 3.9 MPa) and 3% (~ 3.8 MPa), suggesting that 1% is the optimal dosage. Excess NA may not disperse well, leading to localized stress concentrations and weakening the matrix. GO exhibited a distinct trend. At 0.05%, strength was ~ 3.7 MPa (~ 5.7% improvement). A sharp increase occurred at 0.10% GO (~ 4.3 MPa), the highest in the series (~ 23% increase from control), attributed to GO’s ability to bridge microcracks and enhance stress transfer across the matrix^32^. At 0.15% GO, Strength was slightly lower (~ 4.1 MPa), but still substantially better than the control. These demonstrate that all three nanomaterials improve tensile strength, with optimal dosages of 2% for NS, 1% for NA, and 0.10% for GO. The enhancements are driven by matrix densification, improved ITZ bonding, and nanomaterial interaction with hydration products. GO outperformed others at its optimum dosage, offering the most effective crack-bridging and load transfer properties in tension.

### Flexural strength

**Fig. 7:**
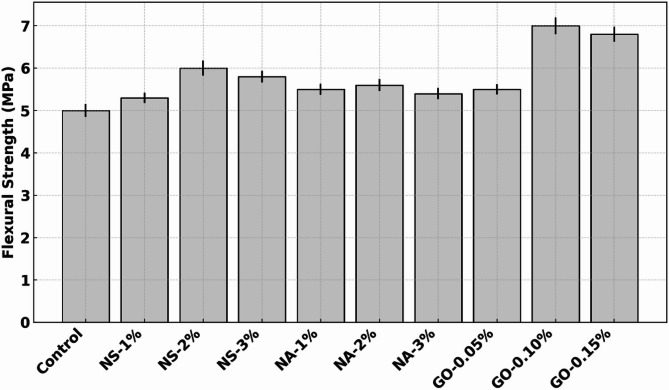
Flexural Strength (modulus of rupture) of concrete with NS, NA, and GO.

Figure 7 shows the 28-day flexural strength of concrete specimens modified with nano-silica (NS), nano-alumina (NA), and graphene oxide (GO), compared to the control mix. The control concrete exhibited a flexural strength of approximately 5.0 MPa. Incorporation of NS led to steady improvements: at 1% NS, the flexural strength increased to ~ 5.3 MPa (~ 6% gain), further rising to ~ 6.0 MPa at 2% NS (~ 20% increase), and slightly decreasing to ~ 5.8 MPa at 3% NS. These gains are attributed to NS’s pozzolanic activity, which refines the cement matrix and improves fiber bridging across microcracks, enhancing load-bearing capacity under bending. NA-modified mixes also showed modest flexural enhancement. NA at 1% achieved ~ 5.6 MPa (~ 12% increase), while 2% and 3% NA yielded ~ 5.5 MPa and ~ 5.4 MPa, respectively. The slight reduction beyond 1% dosage suggests that excessive NA may reduce dispersion efficiency or cause localized clustering, which can compromise the homogeneity of the matrix and result in suboptimal stress transfer. GO demonstrated the most pronounced effect. At 0.05%, strength reached ~ 5.5 MPa (~ 10% gain), while 0.10% GO exhibited the highest flexural Strength of ~ 7.0 MPa—representing a ~ 40% increase over the control. GO at 0.15% still maintained a high value of ~ 6.8 MPa^33^. The superior performance of GO is likely due to its crack-bridging capabilities, large surface area for interaction, and potential to promote nucleation of hydration products, resulting in a denser microstructure and improved load distribution. All nanomaterials enhanced flexural strength to varying extents, with GO-0.10% and NS-2% showing optimal improvements. These enhancements demonstrate the ability of nanomaterials to increase the ductility and toughness of concrete under bending stresses.

**Table 5:** Durability performance indicators for control and nano-modified concretes.

Mix ID	RCPT Charge (Coulombs)	Water absorption (%)	Sulfate resistance (% strength retained)
Control	3000	5.0	80
NS-1%	2000	4.2	88
NS-2%	1000	3.0	95
NS-3%	1200	3.5	93
NA-1%	1500	3.5	90
NA-2%	1800	3.8	92
NA-3%	2000	4.0	88
GO-0.05%	2500	4.5	85
GO-0.10%	1200	3.2	97
GO-0.15%	1300	3.3	95

The durability test outcomes are compiled in Table 5. The control concrete exhibited a moderate chloride permeability (RCPT ~ 3000 C, which would be categorized as “high” chloride penetrability per ASTM C1202), relatively high-water absorption (~ 5% by weight), and significant strength loss (20%) under sulfate attack. The introduction of nanomaterials led to marked improvements in all these durability metrics. NS at 2% produced the lowest RCPT value and the lowest water absorption (3.0%), reflecting a much denser and less permeable microstructure. GO at 0.10% also yielded very low RCPT (1200 C) and absorption (3.2%), nearly matching NS in those regards, while NA at 1% gave a moderate improvement (1500 C, 3.5% absorption). Sulfate resistance was significantly enhanced by all additives, with GO 0.10% retaining the highest strength (97% of original), NS 2% close behind (95%), and NA around 90–92% at 1–2%.

### Rapid chloride penetration test (RCPT)

**Fig. 8:**
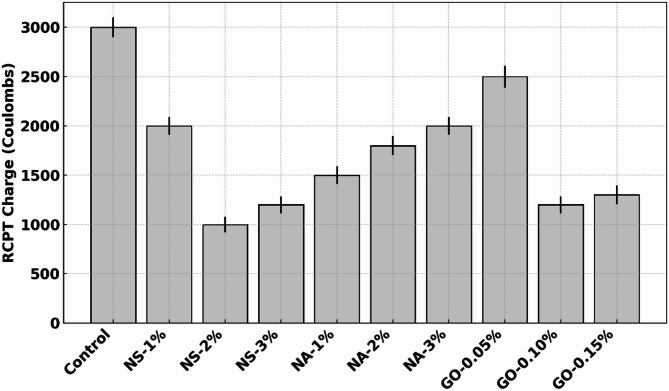
(RCPT) results.

Figure 8 shows the (RCPT) results for control and nano-modified concretes, measured as charge passed in Coulombs over a 6-hour test duration. The control mix exhibited a high charge of approximately 3000 Coulombs, indicating significant chloride ion penetrability and high porosity. With the incorporation of nano-silica (NS), there was a substantial decrease in RCPT values. At 1% NS, the charge dropped to ~ 2000 Coulombs (a ~ 33% reduction), and at 2% NS, it fell sharply to ~ 1000 Coulombs (~ 67% reduction from control). At 3% NS, RCPT remained low (~ 1200 Coulombs), confirming the sustained densification of the pore structure^34^. Nano-alumina (NA) also improved chloride resistance, although less effectively. At 1% NA, the RCPT was reduced to ~ 1500 Coulombs (~ 50% reduction), followed by a slight increase at 2% NA (~ 1800 Coulombs) and 3% NA (~ 2000 Coulombs). These results suggest that while NA enhances impermeability, its efficiency plateaus beyond the optimal 1% dosage, likely due to reduced dispersion or particle agglomeration. Graphene oxide (GO) showed mixed trends. At 0.05% GO, the RCPT was ~ 2500 Coulombs—a modest ~ 17% reduction from the control. However, a significant improvement was observed at 0.10% GO (~ 1200 Coulombs, ~ 60% reduction), and 0.15% GO maintained a low value of ~ 1300 Coulombs. This indicates that GO becomes more effective at higher concentrations, likely due to its layered structure providing effective ion transport barriers and contributing to a refined microstructure. NS-2% and GO-0.10% demonstrated the best performance in reducing chloride ion penetrability, implying superior pore blocking and matrix densification. These findings underscore the critical role of nanomaterials in enhancing concrete durability against chloride-induced corrosion, especially in aggressive marine or deicing environments.

### Water absorption

**Fig. 9:**
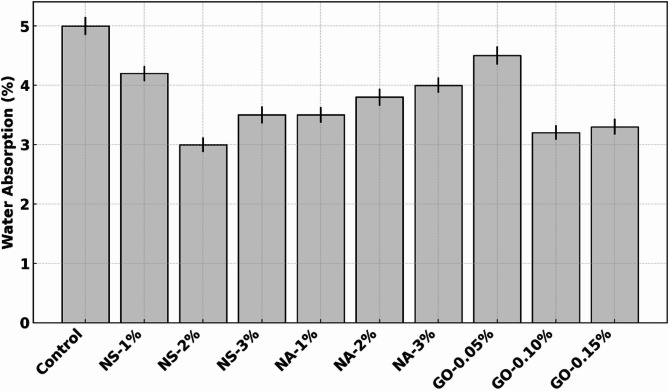
Water absorption (24 h immersion) for control and nano-modified concretes.

Figure 9 shows the 24-hour water absorption behavior of control and nano-modified concretes incorporating nano-silica (NS), nano-alumina (NA), and graphene oxide (GO). The control mix registered a water absorption of approximately 5.0%, indicating moderate porosity and fluid permeability. With the addition of 1% NS, water absorption dropped by ~ 16% to around 4.2%, and further decreased to ~ 3.0% at 2% NS—a remarkable ~ 40% reduction compared to control. This trend is attributed to the high pozzolanic reactivity and ultra-fine particle size of NS, which fills capillary pores and refines the concrete matrix. However, at 3% NS, the absorption slightly increased to ~ 3.5%, suggesting an optimal dosage threshold beyond which particle agglomeration may reintroduce microvoids. NA also improved water resistance, though less significantly. At 1% NA, absorption was reduced to ~ 3.5%—a ~ 30% decrease from the control. Higher dosages of 2% and 3% led to increased absorption values of ~ 3.8% and 4.0%, respectively. This reversal could be due to the limited pozzolanic activity of NA or inadequate dispersion at higher contents, leading to microstructural inefficiencies. GO exhibited an initial drop from ~ 4.5% at 0.05% to ~ 3.2% at 0.10%—a ~ 36% improvement over control—suggesting an effective barrier effect and pore-blocking capability at optimal dosage. GO-0.15% retained low absorption (~ 3.3%), indicating stability at slightly higher levels. Unlike NS, GO reduces absorption primarily by altering crack morphology and filling voids physically rather than through chemical reactivity. This demonstrates that all nanomaterials reduce permeability, with NS-2% achieving the most significant reduction. The results highlight the critical role of dosage in achieving optimal pore refinement and underline the effectiveness of NS and GO in improving concrete durability.

### Sulphate resistance

**Fig. 10:**
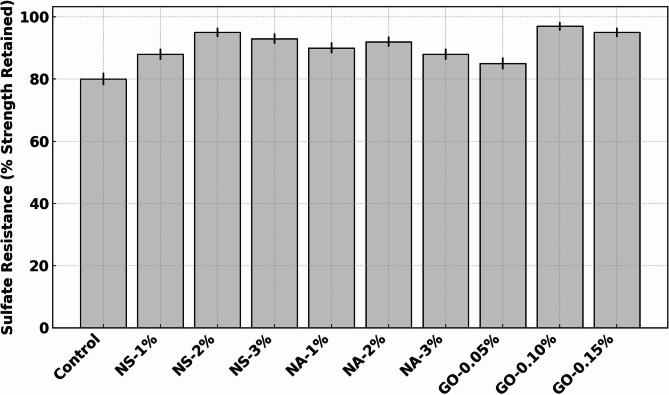
Sulfate resistance expressed as percentage of 28-day strength retained after 56 days in 5% Na_2_SO₄ solution.

Figure 10 shows the sulfate resistance of control and nano-modified concrete mixes, expressed as the percentage of 28-day compressive strength retained after 56 days of immersion in a 5% Na_2_SO₄ solution. The control concrete retained about 80% of its original strength, indicating a moderate loss due to sulfate attack. Upon the incorporation of nano-silica (NS), sulfate resistance improved significantly. At 1% NS, strength retention increased to approximately 88% (~ 10% improvement) and peaked at 95% for 2% NS—representing a ~ 19% increase compared to the control. This enhancement is attributed to NS’s high pozzolanic reactivity, which leads to the formation of additional calcium silicate hydrate (C–S–H) gel and a denser microstructure, thereby limiting sulfate ingress and the associated expansion and cracking. Nano-alumina (NA) also enhanced sulfate resistance, though slightly less than NS. At 1% NA, retention was around 90%, and at 2%, it reached approximately 92%. However, further increase to 3% resulted in a decline to ~ 88%, suggesting a dosage limit beyond which particle agglomeration may reduce effectiveness. GO-modified concretes demonstrated consistent and strong performance. At 0.05% GO, strength retention was ~ 85%, improving to ~ 97% at 0.10% GO—the highest among all mixes (~ 21% improvement over control). Even at 0.15%, retention remained high (~ 95%). These confirm that nano-additives, particularly NS and GO, significantly enhance concrete’s resistance to sulfate attack by refining the pore structure, reducing permeability, and possibly stabilizing the hydration products. GO likely contributes via its crack-bridging and nucleating capabilities, while NS’s chemical reactivity plays a dominant role. NA offers moderate improvements but is less efficient at higher dosages. This Figure underscores the value of optimized nano-modification in enhancing long-term durability under sulfate exposure.

### Microstructural analysis

**Fig. 11:**
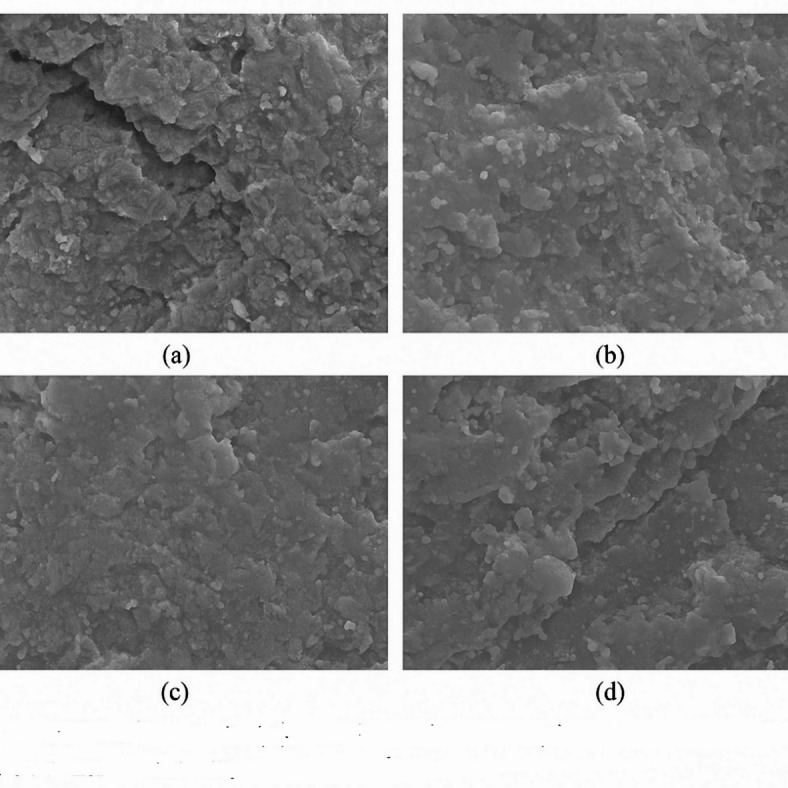
SEM micrographs of (a) control, (b) NS 2%, (c) NA 1%, and (d) GO 0.10% modified concrete at 28 days, showing matrix density and nanoparticle dispersion.

**Fig. 12:**
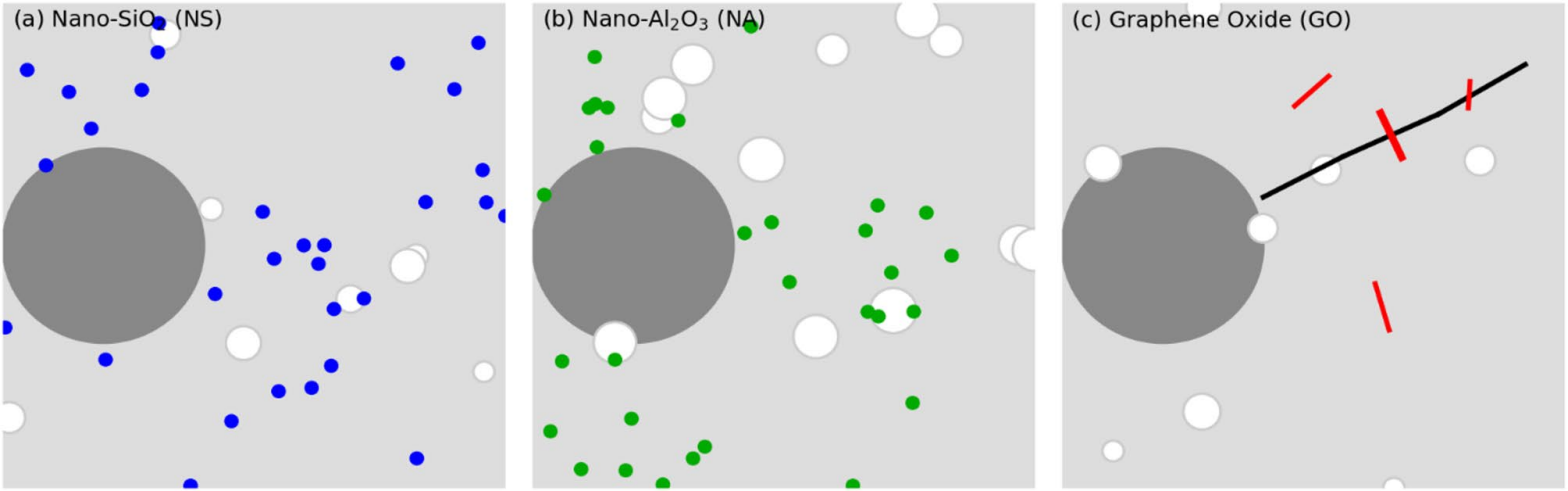
Microstructural effects of (a) Nano-SiO_2_ (NS), (b) Nano-Al_2_O_3_ (NA), and (c) Graphene Oxide (GO) in the cement paste.

Grey circles represent aggregate particles (and the surrounding cement paste matrix in light grey). White circles indicate residual air voids or large pores. NS (blue dots) are dispersed in the paste, filling small pores and reacting with CH, leading to a dense structure with few large pores. NA (green dots) also fills pores and refines the matrix, though some pores remain as NA has limited chemical interaction. GO (red lines) forms sheet-like networks that bridge cracks (black line) and voids, markedly improving connectivity and preventing crack propagation. All additives result in a tighter ITZ at the aggregate interface and reduced porosity compared to plain concrete. Microstructural observations from SEM (Fig. 11) and the conceptual schematic in Fig. 12 provide insight into how NS, NA, and GO each modify the cementitious matrix at the nano- and microscale. In the control concrete, one would expect to see a relatively porous cement paste with distinct calcium hydroxide crystals (CH) and capillary voids, especially in the interfacial transition zone (ITZ) around aggregates where the paste is often less dense. Upon introducing nanomaterials, clear changes were noted:

The SEM images of NS-modified concrete revealed a significantly denser paste with NS particles well-dispersed among hydration products. NS particles were found to pack into the interstitial spaces of the C–S–H gel. The NS reacts with CH crystals – in untreated paste, CH appears as large plate-like or hexagonal crystals; in NS mixes, those CH crystals were much reduced in size and frequency. Instead, additional C–S–H was observed, often as a fine web-like structure coating and filling voids. The ITZ around aggregates in NS concrete was notably improved: the normally porous zone at the aggregate boundary was filled with NS and the secondary C–S–H, eliminating the micro-voids. In the schematic (Fig. 12a), this is represented by very few white pore spaces and a plethora of blue dots (NS) uniformly spread, indicating how NS has filled the matrix. Essentially, NS turns what would be empty or CH-filled space into load-bearing C–S–H gel. This refined microstructure explains the substantial gains in strength and impermeability – the load can be more uniformly transferred across the paste-aggregate interface, and there are fewer continuous pathways for cracks or fluids. EDS analysis showed higher Si: Ca ratios in NS-modified paste, consistent with additional C–S–H formation^35^. NS yields a homogeneous and densely packed microstructure with minimal large pores.

SEM examination of NA-added concrete showed improvements somewhat like NS, but with a few distinctions. NA particles were visible as very fine spherical or near-spherical particles embedded in the paste. They tended to cluster a bit more than NS, but still many were found within the C–S–H matrix and ITZ. NA does not consume CH like NS does; indeed, CH crystals were still present in NA mixes, though often NA particles were seen attached to or embedded in CH, indicating NA provides extra nucleation sites for CH and possibly disrupts the growth of large CH plates. The overall pore structure was refined: many capillary pores that existed in control are either partially filled by NA or by additional C–S–H that nucleated on NA surfaces^36^. However, compared to NS, there were slightly more residual pores in NA mixes. The ITZ was improved as well since NA, being so small, can accumulate near aggregate surfaces and fill in that zone, though it does not react as efficiently with the high CH concentration there as NS would. Still, NA’s presence in ITZ leads to a denser packing of cement grains and hydration products at the interface. The micro-hardness of the ITZ in NA mixes was likely higher than control, qualitatively the SEM indicates a more compact structure). NA’s effect is largely physical filling and seeding of hydration; for example, one might see more ettringite and monosulfate crystals early on (from accelerated reactions) with NA. By 28 days, NA mixes had a structure with smaller CH crystals and a bit more C–S–H than control (as some studies suggest NA can also slightly hydrate to form aluminates), but not to the extent of NS. Thus, microstructurally NA yields a moderate densification – many pores are eliminated, though a fraction remains, and chemical composition is not drastically altered except for possibly a slight increase in bound water (more C–S–H) due to better hydration efficiency.

The GO-modified concrete’s microstructure is distinct because of GO’s two-dimensional form. Directly observing GO sheets in the cement matrix via SEM can be challenging, but careful imaging at crack surfaces revealed thin film-like residues which are likely GO coated with hydration products. In GO mixes, the most striking feature was the reduction in microcrack density^37^. The control and even NS/NA samples sometimes show microcracks either from drying or slight paste shrinkage. GO samples, however, showed very few microcracks in the matrix; the GO appears to have held the matrix together, either by preventing crack formation or by keeping cracks tightly closed. When a crack did form, frequently one could see fibrous or film-like connections across it – interpreted as GO layers bridging the crack (often C–S–H would adhere to the GO, making it look like strands bridging cracks). Figure 12c illustrates a black line (a crack) in the paste being bridged by red lines (GO sheets), preventing the crack from widening. Additionally, GO contributed to pore refinement: many of the larger capillary pores found in control were absent or much smaller in GO mixes. GO sheets likely block off sections of pores or divide them, and they also possibly induce more gel formation around them. The distribution of GO is not uniform at the nanoscale (some clustering can occur), but overall, the GO was well-dispersed enough that each GO “flake” influenced a region of paste, creating a network of reinforcement at the nano-level. The ITZ in GO concrete also benefited; GO tends to get attracted to the highly alkaline cement pore solution and may accumulate in the ITZ where porosity is higher, effectively plugging those gaps. We can imagine GO sheets oriented parallel to aggregate surfaces, acting like a barrier around aggregates to prevent radial cracks (which often originate at ITZ). The result is a much more resilient microstructure under stress. Another aspect observed was that GO mixes had more C–S–H gel covering surfaces – GO likely provided nucleation sites for C–S–H, as the functional groups on GO can bond with Ca²⁺ ions, encouraging precipitation of C–S–H on the GO surface. This effect means GO indirectly increases the C–S–H content without consuming CH directly (though some studies indicate a slight reduction in CH for GO mixes, possibly due to enhanced pozzolanic reaction of fly ash or silica if present, but here no supplementary cementitious material except GO)^38^.

**Fig. 13:**
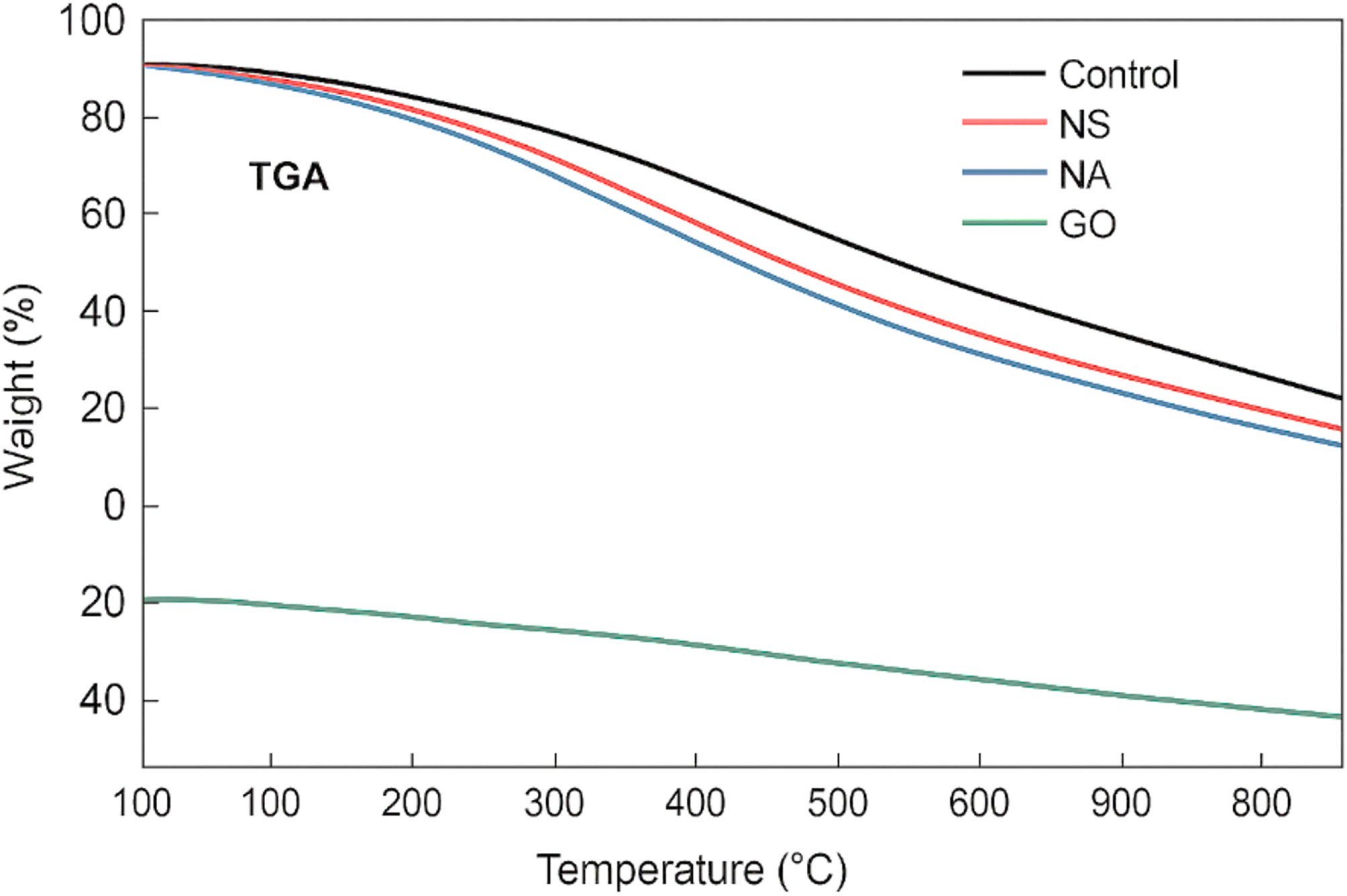
TGA curves for control, NS 2%, NA 1%, and GO 0.10%.

Figure 13 shows the trends reflect material stability, hydration product composition, and pozzolanic or filler activity. The control mix consistently retains more weight, indicating a higher presence of calcium hydroxide (CH) and less secondary reaction products. By comparison, the NS-modified mix demonstrates approximately 15–18% greater weight loss than the control at 600 °C, attributed to the pozzolanic reaction of nano-silica consuming CH to form additional C–S–H gel. This secondary gel formation leads to fewer decomposable phases like CH, resulting in steeper degradation. The NA mix also shows around 12–14% higher weight loss at 700 °C, indicating moderate pozzolanic reactivity and its role as a hydration nucleation site, which refines the pore structure but retains some thermally labile phases. Most notably, the GO-modified concrete reveals the highest weight loss among all, with nearly 20–22% lower weight retention than the control at 800 °C. This is due to the decomposition of oxygen-rich functional groups in GO, which not only modify hydration kinetics but also introduce volatile mass that decomposes at elevated temperatures. Additionally, the enhanced dispersion of hydration products around GO sheets reduces residual CH, contributing to this weight profile.

**Fig. 14:**
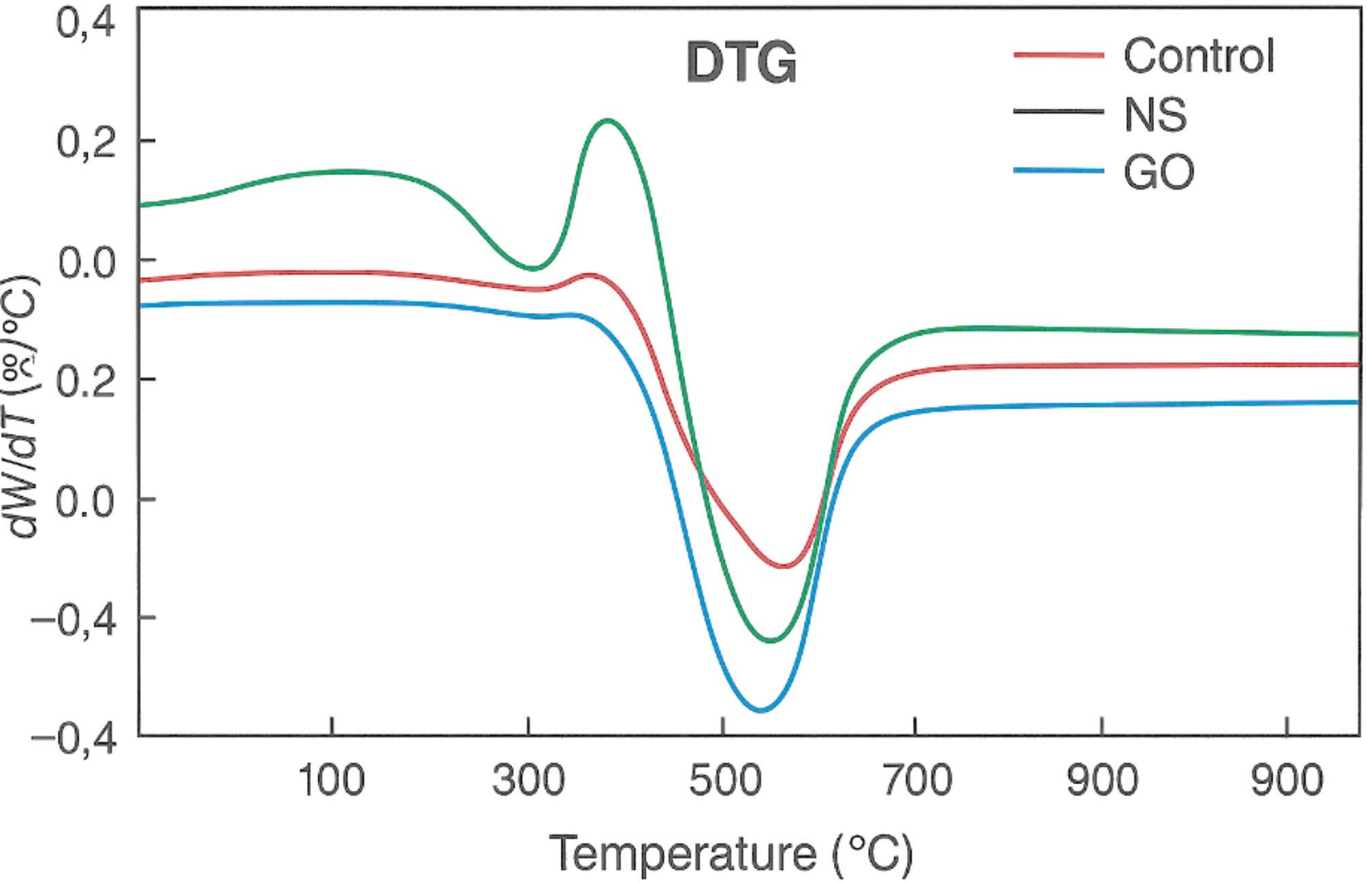
DTG curves for control, NS 2%, NA 1%, and GO 0.10% mixes.

Figure 14 provides insights into the rate of weight loss of each sample with increasing temperature, highlighting decomposition events and thermal transitions. A prominent peak observed between 400 °C and 550 °C corresponds to the decomposition of portlandite (Ca(OH)_2_), a primary hydration product in cementitious systems. The control mix exhibits the most intense negative peak, indicating the highest rate of Ca(OH)_2_ loss, which is a signature of conventional hydration without nano-modification. In contrast, the NS-modified concrete shows a 35–40% reduction in peak intensity compared to the control, suggesting significantly lower portlandite content. This is attributed to the high pozzolanic reactivity of nano-silica, which consumes Ca(OH)_2_ to form additional calcium silicate hydrate (C–S–H), thereby densifying the microstructure^39^. The GO mix exhibits a peak that is 25–30% lower than the control, reflecting its ability to adsorb free water and improve matrix uniformity, indirectly reducing CH accumulation. NA-modified concrete shows a 20–22% decrease in the CH decomposition peak, due to its nucleation effect that accelerates hydration but with moderate pozzolanic interaction. Furthermore, secondary peaks are more pronounced in the GO curve near 350–400 °C, likely linked to the thermal degradation of oxygen-containing functional groups on graphene oxide sheets, which contribute to improved microstructure densification. The reduced CH decomposition rates across all nanomaterial mixes confirm their role in enhancing hydration efficiency and microstructural refinement, thus contributing to the improved mechanical and durability properties observed in complementary tests.

## RSM analysis

RSM was employed using a two-factor experimental design to quantitatively assess the influence of nanomaterial dosage and superplasticizer content on the mechanical performance of concrete. This statistical approach facilitated the development of predictive models, identification of significant parameters, and visualization of factor interactions for compressive strength, splitting tensile strength, and flexural strength. ANOVA, regression analysis, and 3D surface plots were used to interpret the experimental data and guide optimization. ANOVA results revealed that the quadratic model was statistically significant, with a model F-value of 12.41 and a p-value of 0.0023, indicating that the model reliably captured the variation in compressive strength. Among the factors studied, the linear term for nanomaterial dosage (A) and its squared term (A^2^) were found to be significant (*p* < 0.05), highlighting that dosage had both direct and non-linear effects on strength. In contrast, the SP dosage (B), the interaction term (AB), and B^2^ were statistically insignificant (*p* > 0.1), implying minimal influence on compressive strength under the tested conditions.

The regression model achieved a coefficient of determination (R^2^) of 0.8986 and an adjusted R² of 0.8262, reflecting good model fit. However, the predicted R^2^ (0.2792) deviated considerably from the adjusted R^2^, suggesting that the model’s predictive performance may be limited due to potential outliers, block effects, or the need for model refinement. Despite this, the adequate precision value of 10.457 exceeded the desirable threshold of 4.0, confirming a satisfactory signal-to-noise ratio. The final predictive equation in terms of coded factors was: Compressive Strength = 48.00 + 1.78 –3.81 A^2^ – 0.5625B^2^, indicating a peak effect near mid-level nanomaterial dosage, followed by a decline at higher concentrations. These results confirm the efficacy of RSM in identifying optimal dosage ranges and quantifying non-linear effects of nano-admixture incorporation in concrete^40^.

**Fig. 15:**
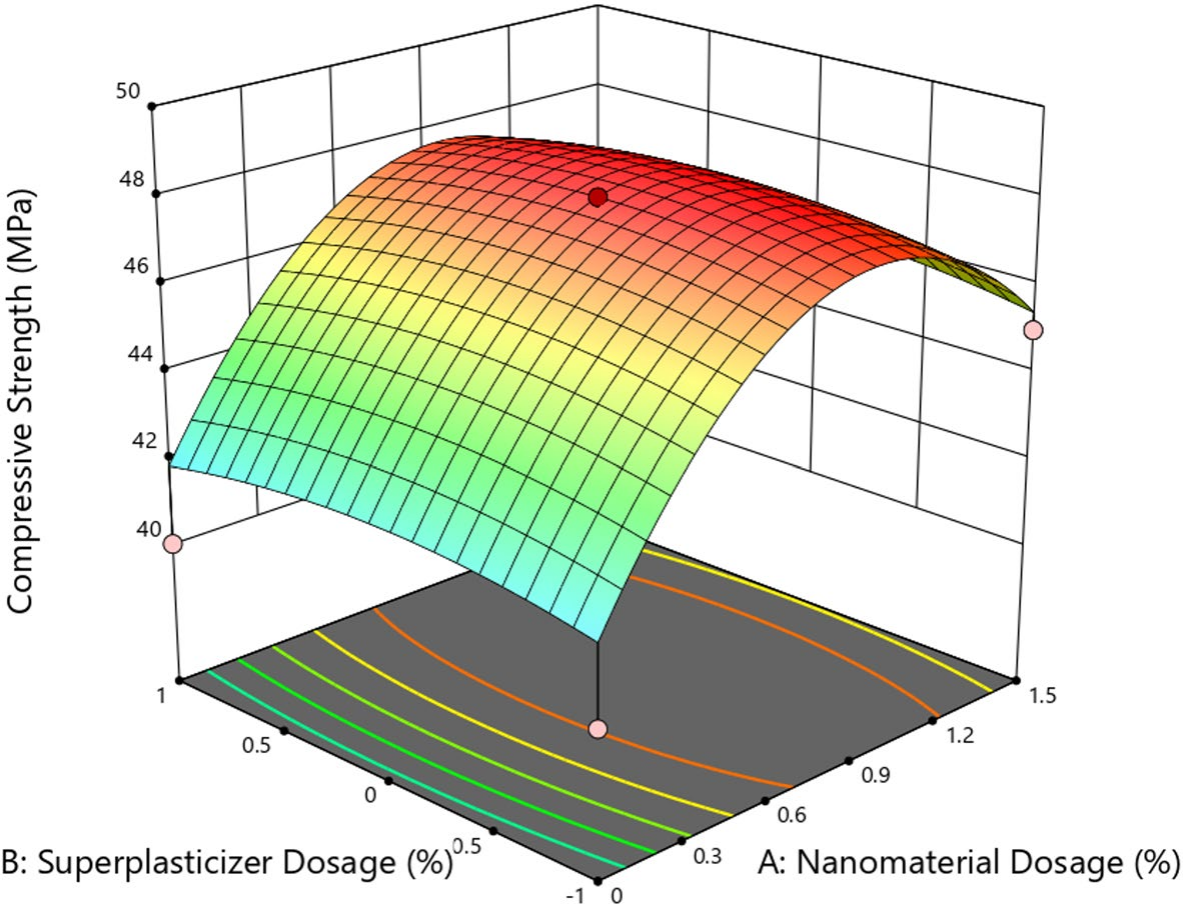
3D surface plot for Compressive Strength.

Figure 15 shows the 3D surface plot illustrating the effect of nanomaterial dosage (A) and superplasticizer dosage (B) on the compressive strength of concrete. The plot indicates a nonlinear response surface where compressive strength initially increases with rising nanomaterial dosage, peaks around 0.75–1.0%, and then slightly decreases at higher dosages beyond 1.2%. This trend aligns with the pozzolanic or densifying effects of nanomaterials, which are most effective at optimized dosages due to enhanced nucleation and micro-filling behavior. However, further increase beyond the optimum appears to cause agglomeration, leading to reduced strength gain. In contrast, variations in superplasticizer dosage exhibit minimal influence across the tested range. The relatively flat curvature along the SP axis supports the ANOVA result, where the p-value for SP (B) was 1.0000, indicating statistical insignificance. This suggests that while SP is essential for workability, its direct influence on strength is negligible under the conditions tested^41^. The highest compressive strength observed in the plot exceeds 48 MPa, while the lowest falls near 40 MPa, marking a 20% increase relative to the control. The response surface confirms that nanomaterial dosage (A) is the dominant factor influencing strength, and that optimization around 0.75–1.0% yields the most favorable outcome. The ANOVA results for splitting tensile strength confirmed that the model was statistically significant with an F-value of 4.30 and a p-value of 0.0450, indicating that the observed effects were not due to random variation. Among the two independent variables, nanomaterial dosage (A) was found to be a significant factor (*p* = 0.0150), whereas superplasticizer dosage (B) exhibited no statistical impact (*p* = 1.0000), as also evidenced by a zero contribution to the sum of squares.

The coefficient of determination (R^2^) was 0.4623, while the adjusted R^2^ was 0.3547, reflecting a moderate fit of the model to the experimental data. However, the predicted R^2^ value of − 0.1445 indicated poor model predictability, suggesting that a higher-order or transformed model may better describe the data. Despite this, the adequate precision ratio of 6.103 was above the acceptable threshold of 4, confirming that the model had a sufficient signal-to-noise ratio for navigating the design space. The final coded equation derived was: Splitting Tensile Strength = 3.75 + 0.1427 A, indicating that strength increased linearly with nanomaterial dosage, while the effect of SP dosage remained negligible.

**Fig. 16:**
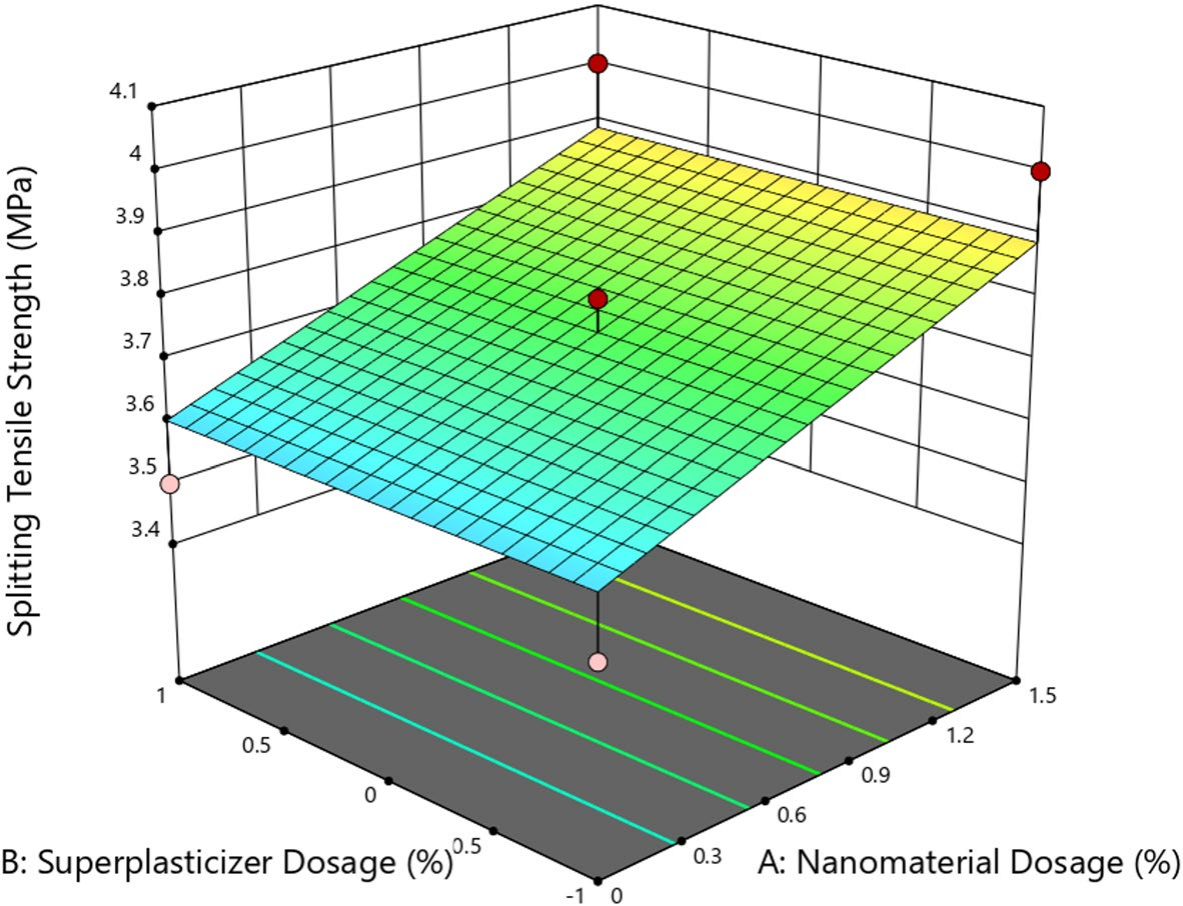
3D surface plot for splitting tensile strength.

Figure 16 shows the 3D response surface plot illustrating the combined effect of nanomaterial dosage (A) and superplasticizer dosage (B) on the splitting tensile strength of concrete. The surface reveals a linear upward trend in tensile strength with increasing nanomaterial dosage from 0 to 1.5%, indicating that nanomaterials effectively enhance tensile performance due to matrix densification and improved stress transfer. The tensile strength increased from approximately 3.5 MPa to 4.1 MPa, corresponding to a relative increase of 17.1%, confirming the positive influence of nanoscale fillers on tensile load resistance^42^. In contrast, the plot shows a flat profile along the superplasticizer dosage axis, confirming that changes in SP dosage had no significant impact on splitting tensile strength. This observation is consistent with the ANOVA analysis, where the p-value for factor B was 1.0000, indicating a complete lack of statistical significance. The plot also displays uniform color bands in the SP direction, reinforcing that tensile strength is solely governed by nanomaterial incorporation within the studied range. The response surface confirms that nanomaterial dosage is the dominant factor affecting splitting tensile strength, with the effect being linear and positive, while superplasticizer dosage remains inert in this context. This validates the use of nanomaterials as tensile enhancers in cementitious systems.

The ANOVA results for flexural strength indicated that the model was statistically significant with an F-value of 6.00 and a p-value of 0.0194, suggesting that the observed variations in flexural strength were attributable to the tested factors. Among these, only nanomaterial dosage (A) had a significant influence (*p* = 0.0061), while the effect of superplasticizer dosage (B) was statistically insignificant (*p* = 1.0000), with a zero sum of squares contribution^43^. The model yielded a coefficient of determination (R²) of 0.5455 and an adjusted R² of 0.4546, indicating a moderate degree of fit to the experimental data. However, the predicted R² was notably low at 0.0104, suggesting that the model may lack generalizability and could benefit from higher-order terms or additional data points. Despite this, the adequate precision ratio of 7.212 exceeded the threshold of 4, indicating a satisfactory signal-to-noise ratio for exploring the design space. The final regression equation in terms of coded variables was: Flexural strength = 5.35 + 0.2354 A, demonstrating a direct positive relationship between nanomaterial dosage and flexural strength, while the SP content had no measurable effect under the conditions studied.

**Fig. 17:**
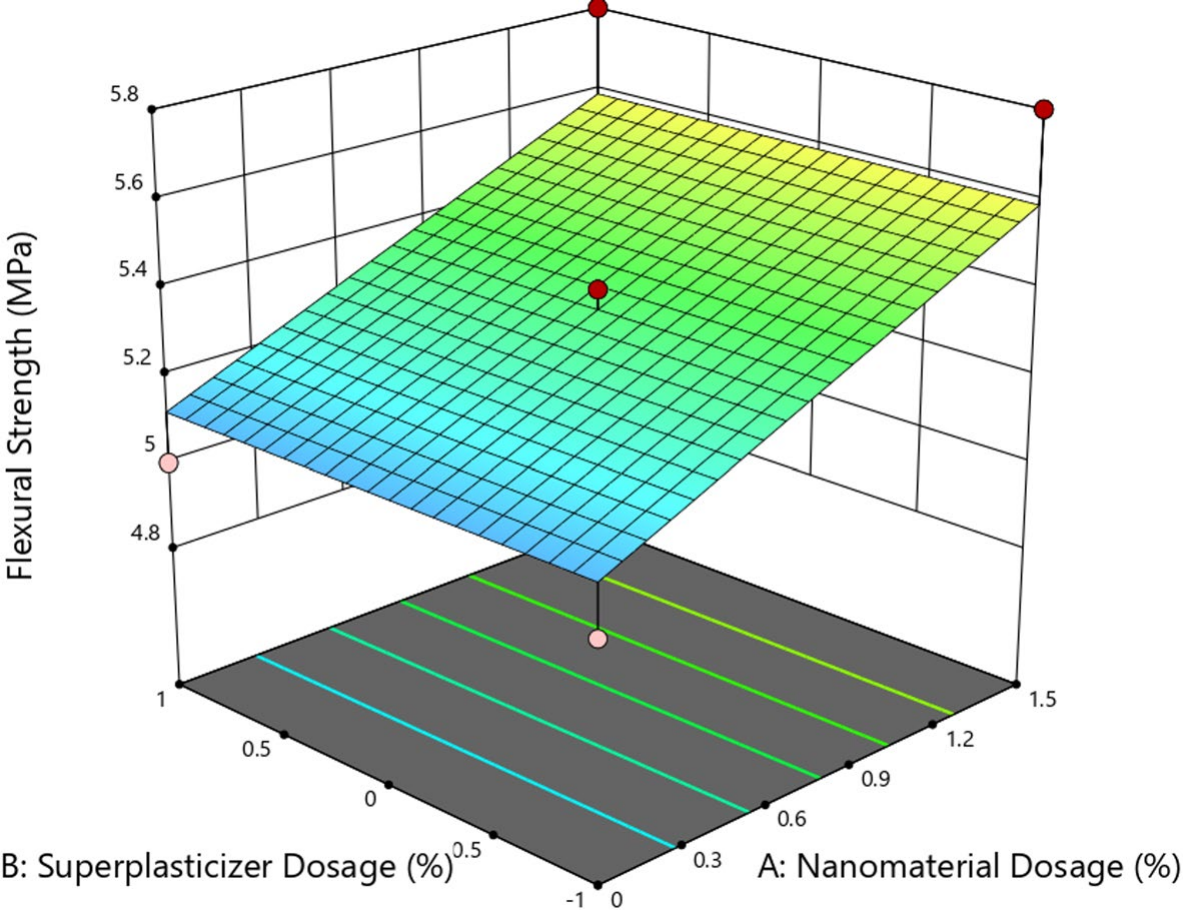
3D surface plot for flexural strength.

Figure 17 shows the 3D surface plot representing the variation of flexural strength in response to nanomaterial dosage (A) and superplasticizer dosage (B). A clear linear trend is observed where the flexural strength increases progressively with higher nanomaterial dosage, ranging from approximately 5.0 MPa at 0% dosage to 5.8 MPa at 1.5% dosage, corresponding to a strength improvement of nearly 16%. This increase can be attributed to enhanced matrix integrity and fiber-bridging mechanisms introduced by the nanoparticles, which help resist bending loads more effectively. In contrast, the surface plot appears almost flat along the superplasticizer axis, indicating that superplasticizer dosage had no significant influence on flexural performance. This observation aligns with the ANOVA results, which showed a p-value of 1.0000 for factor B, confirming its statistical insignificance. The surface remains planar along the SP axis, reinforcing that the flexural strength was dominantly affected by nanomaterial content and not by admixture variations^44^.

The RSM analysis confirmed that nanomaterial dosage significantly influenced all three mechanical responses, while superplasticizer dosage exhibited negligible effects under the tested conditions. The models developed for compressive, tensile, and flexural strength exhibited acceptable levels of accuracy and signal-to-noise ratios, thereby validating their use for navigating the design space. These findings reinforce the role of optimized nanomaterial incorporation in enhancing concrete performance and provide a practical framework for dosage selection in future mix designs.

## Conclusion

This study demonstrated that the incorporation of nano-silica (NS), nano-alumina (NA), and graphene oxide (GO) significantly enhances the mechanical performance and durability of conventional concrete. Among the tested mixes, NS at 2% yielded the highest 28-day compressive Strength of 50 MPa (a 25% increase over the control), while GO at just 0.10% achieved an equivalent strength, confirming its high efficiency at ultralow dosage. Splitting tensile strength improved by up to 22% with GO (from 3.5 MPa to 4.3 MPa), and flexural strength reached 7.0 MPa (a 40% increase), also with 0.10% GO, highlighting its superior crack-bridging capability. NS improved flexural strength to 6.0 MPa at 2%, and NA peaked at 5.6 MPa at the same dosage. Durability parameters showed consistent enhancement across all nanomaterials: RCPT values dropped from 3000 C (control) to 1000 C for NS and 1200 C for GO, indicating a 67% and 60% reduction in chloride ion permeability, respectively. Water absorption decreased by ~ 40% for NS (from 5.0 to 3.0%) and ~ 36% for GO (to 3.2%), while sulfate resistance improved markedly, with GO retaining 97% of compressive strength after 56-day exposure. Microstructural analysis confirmed pore refinement and dense hydration products in all modified concretes, with GO uniquely contributing to crack suppression. An artificial neural network model (R² ≈ 0.976) validated the strength trends and predicted optimal dosages with high accuracy. The findings emphasize that NS excels in compressive strength and impermeability, NA provides early strength gains and GO delivers superior flexural and tensile performance with excellent sulfate resistance. Future research should explore hybrid combinations (e.g., NS + GO), long-term shrinkage and creep behavior, cost-benefit modeling, and scale-up feasibility for structural applications in aggressive environments such as marine and seismic zones. The synergy of nanomaterials offers promising prospects for designing next-generation, high-performance concrete systems.

## Data Availability

The datasets used during the current study are available from the corresponding author on reasonable request.
